# Development and Genetic Characterization of Peanut Advanced Backcross Lines That Incorporate Root-Knot Nematode Resistance From *Arachis stenosperma*

**DOI:** 10.3389/fpls.2021.785358

**Published:** 2022-01-17

**Authors:** Carolina Ballén-Taborda, Ye Chu, Peggy Ozias-Akins, C. Corley Holbrook, Patricia Timper, Scott A. Jackson, David J. Bertioli, Soraya C. M. Leal-Bertioli

**Affiliations:** ^1^Institute of Plant Breeding, Genetics and Genomics, University of Georgia, Athens, GA, United States; ^2^Department of Horticulture, University of Georgia, Tifton, GA, United States; ^3^U.S. Department of Agriculture-Agricultural Research Service (USDA-ARS), Tifton, GA, United States; ^4^Department of Crop and Soil Science, University of Georgia, Athens, GA, United States; ^5^Department of Plant Pathology, University of Georgia, Athens, GA, United States

**Keywords:** wild crop relatives, *Arachis*, peanut, root-knot nematode, *Meloidogyne arenaria*, marker-assisted backcrossing, domestication

## Abstract

Crop wild species are increasingly important for crop improvement. Peanut (*Arachis hypogaea* L.) wild relatives comprise a diverse genetic pool that is being used to broaden its narrow genetic base. Peanut is an allotetraploid species extremely susceptible to peanut root-knot nematode (PRKN) *Meloidogyne arenaria*. Current resistant cultivars rely on a single introgression for PRKN resistance incorporated from the wild relative *Arachis cardenasii*, which could be overcome as a result of the emergence of virulent nematode populations. Therefore, new sources of resistance may be needed. Near-immunity has been found in the peanut wild relative *Arachis stenosperma*. The two loci controlling the resistance, present on chromosomes A02 and A09, have been validated in tetraploid lines and have been shown to reduce nematode reproduction by up to 98%. To incorporate these new resistance QTL into cultivated peanut, we used a marker-assisted backcrossing approach, using PRKN *A. stenosperma*-derived resistant lines as donor parents. Four cycles of backcrossing were completed, and SNP assays linked to the QTL were used for foreground selection. In each backcross generation seed weight, length, and width were measured, and based on a statistical analysis we observed that only one generation of backcrossing was required to recover the elite peanut’s seed size. A populating of 271 BC_3_F_1_ lines was genome-wide genotyped to characterize the introgressions across the genome. Phenotypic information for leaf spot incidence and domestication traits (seed size, fertility, plant architecture, and flower color) were recorded. Correlations between the wild introgressions in different chromosomes and the phenotypic data allowed us to identify candidate regions controlling these domestication traits. Finally, PRKN resistance was validated in BC_3_F_3_ lines. We observed that the QTL in A02 and/or large introgression in A09 are needed for resistance. This present work represents an important step toward the development of new high-yielding and nematode-resistant peanut cultivars.

## Introduction

*Arachis hypogaea* L., with a common name of peanut or groundnut, is an important oil, food, and fodder crop cultivated worldwide with an annual production of 66.3 million tons and grown on 34.1 Mha (FAOSTAT, 2021). Peanut is an allotetraploid species (AABB, 2n = 4x = 40), with a recent and unique polypoid origin, which occurred 5 to 10 thousand years ago (Bertioli et al., 2019, 2020). This narrow genetic base and limited gene flow with its genetically diverse diploid wild relatives resulted in a lack of strong resistance alleles for pests and diseases in the primary gene pool. One important pest is the peanut root-knot nematode (PRKN) (*Meloidogyne arenaria*) (Holbrook and Stalker, 2003). It causes yield losses greater than 50% in infested fields, and at times, 100% losses in heavily infested areas of fields have been reported (Dickson and De Waele, 2005; Timper et al., 2018). In the United States, *M. arenaria* is the most damaging nematode for peanut (Timper et al., 2018). Chemical control is one option, but is costly, hazardous to human health, and can damage the environment (Oka, 2020). Crop rotation is also effective, but with susceptible cultivars, the constraints on the frequency at which peanut can be grown reduce agronomic and financial sustainability. The use of high-yielding and nematode-resistant cultivars in combination with rotation is the most efficient and effective way to control nematode populations and maintain yield while reducing the use of nematicides.

Strong resistance to many pests and diseases is limited in the *A. hypogaea* primary gene pool (Stalker, 2017), which imposes constraints for crop improvement using cultivated germplasm (Nelson et al., 1989; Noe et al., 1992). Yet, the wild relatives comprise a diverse genetic pool that has the potential to broaden a peanut’s genetic base and to improve its performance under pest/disease pressure (Stalker, 2017). Previously, successful transfer of root-knot nematode resistance into cultivated peanut was accomplished through backcrossing schemes involving a synthetic allotetraploid (Simpson and Starr, 2001). This resistance is derived from introgression of a large segment on chromosome A09 from the wild species *Arachis cardenasii* (Nagy et al., 2010; Chu et al., 2016), and is present in several commercial cultivars (Georgia-14N, TifNV-High O/L, Tifguard, NemaTAM, and Webb) (Simpson et al., 2003, 2013; Holbrook et al., 2008, 2017; Branch and Brenneman, 2015). Although this resistance has been durable thus far, occasional resistance breakdown has been reported (Holbrook CC, personal communication). Therefore, it is important to incorporate new sources of resistance to reduce the risk of selection of a virulent population of *M. arenaria* and to guarantee continued protection of the peanut crop from losses due to PRKN.

The peanut wild relative *Arachis stenosperma* PI666100/V10309 has been described as highly resistant to peanut root-knot nematode (Proite et al., 2008). Previously, three quantitative trait loci (QTL) (on chromosomes A02, A04, and A09) were identified in the diploid genome of *Arachis stenosperma* (Leal-Bertioli et al., 2016). Later, segments of both chromosomes A02 and A09, that provide near immunity, were mapped using a segregating population derived from a cross between *A. hypogaea* and the synthetic allotetraploid BatSten1 (Bertioli et al., 2021a), and validated in a tetraploid background (Ballén-Taborda et al., 2019, 2021). The main objective of this study was to incorporate PRKN resistance QTL from *A. stenosperma* into elite peanut. To accomplish this goal, a marker-assisted backcross breeding approach was employed and BatSten1 was used as the donor parent. Four cycles of backcrossing were completed with genetic foreground and background selection and phenotypic characterization were performed in each generation. Correlations between the wild introgressions across the genome and the phenotypic data allowed us to identify candidate regions controlling traits measured in the BC_3_F_1_ population.

This work is key to developing new high-yielding peanut cultivars with a new and strong resistance against the peanut root-knot nematode. Additionally, single nucleotide polymorphism (SNP) markers tightly linked to the QTL are described to facilitate the introgression of *A. stenosperma* resistance into different elite recipient lines. In the near future, we expect to release advanced introgression lines that incorporate strong PRKN resistance with attached molecular information, that can be used directly in breeding programs in areas where PRKN is a constraint for peanut cultivation.

## Materials and Methods

### Plant Materials

**FIGURE 1:**
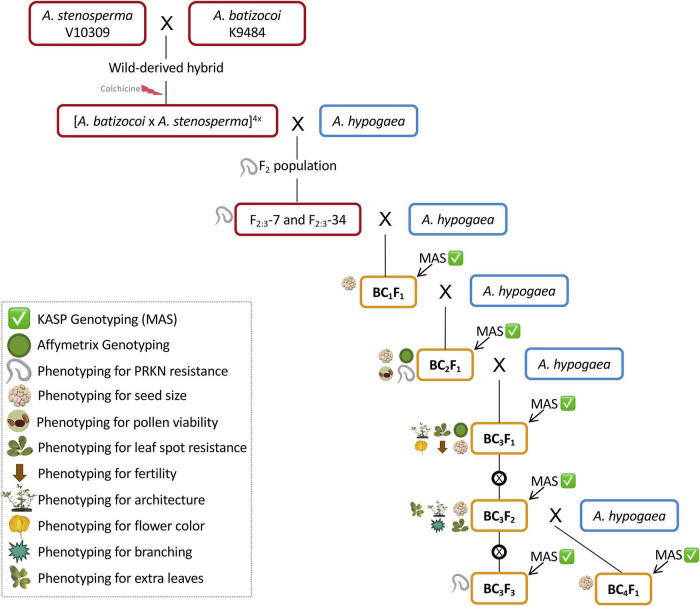
Schematic of the marker-assisted backcrossing scheme for incorporation of peanut root-knot nematode resistance from the wild species *A. stenosperma* V10309 into peanut breeding lines from Tifton, GA. Superior F_2_-derived F_3_ lines (Ballén-Taborda et al., 2019) were selected as donor parents (DP, red boxes). Recurrent parents (RP, blue boxes) included TifGP-2, 5-646-10, 13-1014, 13-2113, and 13-1125. In each cycle, KASP genotyping was performed to identify lines carrying resistance loci in chromosomes A02 and A09 (Marker-assisted selection – MAS) (green checkmark) (Supplementary Tables 1, 2, gray-shaded markers). BC_*n*_F_*n*_ progeny from each cycle were used as male parents for the next (orange boxes). All BC_*n*_F_*n*_s were genotyped for seed size (weight, length, and width) (seed symbol). BC_2_F_1_s were genome-wide genotyped and phenotyped for PRKN resistance for validation (green circle and nematode symbol) (Ballén-Taborda et al., 2021) and pollen viability was studied (pollen symbol). BC_3_F_1_s were subjected to genome-wide genotyping for characterization of introgressions (green circle) and phenotyped for leaf spot incidence, fertility, architecture, and flower color (indicated by leaf spot, brown arrow, architecture, and flower symbols, respectively). BC_3_F_2_s were phenotyped for leaf spot incidence, architecture, branching, and extra leaves (indicated by leaf spots, architecture, branching, and leaves symbols, respectively). BC_3_F_3_s were phenotyped for PRKN resistance and KASP genotyped (green checkmark and nematode symbol). Self-pollination is represented by ⊗.

The synthetic allotetraploid BatSten1 PI 695418 {[*Arachis batizocoi* PI298639/K9484 x *A. stenosperma* PI666100/V10309]^(2*n* = 4*x* = 40)^} (Bertioli et al., 2021a) was used to introgress the nematode resistance QTL from *A. stenosperma* into tetraploid peanut. An F_2_ population was created by selfing an F_1_ derived from a cross between *A. hypogaea* cv. Runner IAC-886 (herein called Runner-886) and BatSten1, and then used for QTL mapping (Ballén-Taborda et al., 2019). From this population, four superior F_2_ lines (F_2_-7, F_2_-13, F_2_-34, and F_2_-73) were selected based on (1) better vigor, as visually more leaf biomass; (2) good agronomic traits; (3) late leaf spot (LLS) and PRKN resistance; (4) harboring QTL in A02 and A09 for nematode resistance per molecular genotyping. Six F_2_-derived F_3_ (F_2:3_) homozygous progeny from F_2:3_-7 and F_2:3_-34, with validated resistance to PRKN (Ballén-Taborda et al., 2021), were used as initial donor parents in a backcrossing scheme (Figure 1, red boxes). For incorporation of resistance from *A. stenosperma* into peanut, we used three susceptible recurrent parents, which are TifGP-2, 5-646-10, and 13-1014. For pyramiding resistances from both wild species *A. stenosperma* and *A. cardenasii*, we used two resistant lines, which are 13-2113 and 13-1125. (1) TifGP-2 is a breeding line with good yield and grade, and normal oleic content (Holbrook et al., 2012); (2) 5-646-10 is derived from the cross Florida-07 x Tifguard, with good yield and grade, and high oleic/linoleic fatty acid ratio (Holbrook, CC, unpublished data); (3) 13-1014 is derived from [C1805-617-1 (Florida-07 x Tifguard) x GA-06G], with high oleic/linoleic fatty acid ratio (Holbrook, CC, unpublished data); (4) 13-2113 is derived from [C1805-2-9-16 (Florida-07 x Tifguard) x TifGP-2], a high oleic/linoleic fatty acid ratio, that was included in the second cycle only (Holbrook, CC, unpublished data); (5) 13-1125 breeding line was included in the fourth cycle only (Holbrook, CC, unpublished data) (Figure 1, blue boxes).

### Marker-Assisted Breeding

A marker-assisted backcrossing (MABC) approach was used to incorporate PRKN resistance from *A. stenosperma* into cultivated peanut. Four cycles of backcrossing were performed in two different locations: Athens, GA and Tifton, GA under greenhouse conditions. In the first, second, and third cycles, 16 SNP markers linked to the QTL in A02 and A09 (Leal-Bertioli et al., 2016) were used for foreground selection (Supplementary Table 1, gray-shaded markers). For the fourth cycle, 10 new markers were used to finely target these chromosome segments based on high-throughput genotyping of backcross (BC) lines (Ballén-Taborda et al., 2021) (Supplementary Table 2, gray-shaded markers). 60 additional SNPs markers were also developed and are available here for genotypic selection in breeding programs (Supplementary Tables 1, 2, not shaded markers). Progeny from each cycle that harbored segments associated with PRKN resistance were used as male parents for the next backcross cycle (Figure 1, orange boxes). BC_4_F_1_s were germinated for seed increase.

Kompetitive Allele-Specific PCR assays (KASP, LGC Biosearch Technologies, Hoddesdon, United Kingdom)^1^ assays were used for the selection of PRKN resistance alleles. For KASP reactions, genomic DNA was isolated from a small section of the peanut cotyledon opposite to the embryo (seed chip, ∼50-100 mg). DNA was extracted using the DNeasy Plant Mini Kit (QIAGEN, Hilden, DE) according to the manufacturer’s instructions. Each KASP marker consisted of three primers per SNP position (two allele-specific and one common flanking primer). Primers were designed using the web-based program BatchPrimer3^2^ (USDA-ARS, Albany, CA, United States) (You et al., 2008) with the “Allele-specific primers and allele flanking primers” option. Parameters used were 60–120 bp in fragment size, GC content of 30–80%, and Tm between 58 and 60°. KASP primer assay mix per SNP position consisted of 12 ul (100 uM) of each allele-specific primer, 30 ul (100 uM) of the flanking primer, and 46 ul of H_2_O. Single KASP reactions (5 ul) consisted of 2.5 ul of KASP 2x Master Mix (Low Rox 5000 V4.0), 0.07 ul of KASP primer assay mix, 1.93 ul of water, and 0.5 ul of DNA (10 ng/ul). Two replicates per primer per sample were included in each reaction, as well as no-template controls (NTCs). A C100 touch Thermal Cycler (BIO-RAD, Hercules, CA, United States) was used with the following conditions: 94°C for 15 min; 8 cycles of 94°C for 20 s and touchdown starting at 61°C for 1 min (dropping 6° per cycle); 31 cycles of 94°C for 10 s and 55°C for 1 min; 9 cycles of 94°C for 20 s, and 57°C for 1 min; 4°C hold. Fluorescence was read with a LightCycler^®^ 480 Instrument II (Roche Life Science, Switzerland) and analyzed using the LightCycler^®^ 480 Software (v.1.5.1.62) (Roche Life Science, Switzerland). Finally, data were exported into Microsoft Excel for analysis.

### Genome-Wide Genotyping of BC_3_F_1_s

Genomic DNA of 271 BC_3_F_1_ lines and controls (BatSten1, Runner-886, 5-646-10, 13-1014, TifGP-2, Tifguard, and Tifrunner) were extracted from lyophilized leaves using the DNeasy 96 Plant Kit (QIAGEN, Hilden, DE, United States) according to the manufacturer’s instructions. DNAs were genotyped with the Axiom_Arachis2 SNP array (Clevenger et al., 2018; Korani et al., 2019). Genotypic data was extracted and processed using the Axiom™ Analysis Suite software (v.4.0.3.3) (ThermoFisher Scientific, Waltham, MA, United States). Output was analyzed using custom shell scripts (see below) and resulting data were visualized as a color map in Microsoft Excel. The physical positions of the A-genome markers were determined according to the position of their orthologs in the *A. duranensis* pseudomolecules and the K-genome markers based on the *A. ipaensis* pseudomolecules (Bertioli et al., 2016).

The strategy to identify polymorphic SNP markers included two main steps. First, a set of polymorphic SNP markers between parental genotypes (BatSten1 ≠ Runner-886) was extracted. Original genotyping calls were replaced by numbers (“1” for BatSten, “2” for Runner-886, and “3” for a different genotype). Second, SNPs present in the genetic map previously identified (*A. stenosperma*-specific and *A. batizocoi*-specific markers) (Ballén-Taborda et al., 2019) were retrieved (Supplementary Script 1). Genotypic data were used to perform the principal component analysis (PCA) using the “*dist*” function in R.

### Peanut Root-Knot Nematode Resistance Validation Using BC_3_F_3_s

BC_3_F_3_ segregating lines from five BC_3_F_1_ families and controls were evaluated for PRKN resistance to further validate the QTL in A02 and/or A09 (Table 1). The experiment included 72 BC_3_F_3_ lines, which were selected based on the genotypic information of the BC_3_F_1_ generation, by focusing on high cultivar genome recovery (89.1–95.9%) and with superior field performance (data not shown) of the BC_3_F_2_ generation during summer 2020. Selected lines included two BC_3_F_3_ lineages with the A02-QTL, two with the A09-QTL, one with both A02-QTL and A09-QTL, and one with a large A09-QTL. The synthetic allotetraploid BatSten1 and the cultivar *A. hypogea* TifNV-High O/L (Holbrook et al., 2017) were used as resistant controls and *A. hypogea* 5-646-10 and 13-1014 as susceptible controls. To confirm the presence of the QTL, BC_3_F_3_s and controls were genotyped using KASP markers (as described in the “Marker-assisted breeding” section). Six KASP markers targeting the bottom of A02 (A02-83,464,195, A02-92,077,207, and A02-92,983,792) and A09 (A09-16,516,448, A09-112309,231 and A09-114,515,959) (Supplementary Table 2) were used. Additionally, Axiom_Arachis2 SNP array genotyping was performed for genome-wide characterization, and data were filtered as described above.

**TABLE 1:** Average and standard deviation for GI/g and eggs/g measured in BC_3_F_3_ lines, resistant, and susceptible controls using a pot bioassay.

Genotype (short)	Type	PRKN resistance segment*	GI/g (avg ± SD)	Eggs/g (avg ± SD)
BatSten1	Resistant control	All segments	0.00 ± 0.00 (d)	0.00 ± 0.00 (b)
*A. hypogaea* TifNV-High O/L (TifNV-O/L)	Resistant control**	-	0.03 ± 0.08 (cd)	6.66 ± 17.43 (b)
*A. hypogaea* 5-646-10 (5-646-10)	Susceptible control	-	0.31 ± 0.26 (ab)	579.18 ± 855.26 (ac)
*A. hypogaea* 13-1014 (13-1014)	Susceptible control	-	0.42 ± 0.11 (b)	1268.64 ± 1046.49 (a)
14_BC_3_F_3_:C2633-2_3(16) S14_B (BC_3_F_3__14_B)	Backcross line – BC_3_F_3_	Bottom A02 (A02)	0.00 ± 0.00 (cd)	30.71 ± 75.23 (b)
170_BC_3_F_3__BRD_C0050_Seed9_S77_A (BC_3_F_3__170_A)	Backcross line – BC_3_F_3_	Bottom A02 (A02)	0.02 ± 0.04 (cd)	11.89 ± 25.08 (b)
133_BC_3_F_3__RBS_sd20_S3_01 (BC_3_F_3__133)	Backcross line – BC_3_F_3_	Bottom A09 (A09)	0.35 ± 0.18 (ab)	1411.04 ± 1076.60 (a)
135_BC_3_F_3__RBS_sd20_S3_03 (BC_3_F_3__135)	Backcross line – BC_3_F_3_	Bottom A09 (A09)	0.25 ± 0.17 (a)	946.29 ± 888.79 (a)
202_BC_3_F_3__RBS_sd14_S1_B (BC_3_F_3__202_B)	Backcross line – BC_3_F_3_	Bottom Small A02 (A02-) and Bottom A09 (A09)	0.13 ± 0.19 (ac)	34.92 ± 73.71 (bc)
203_BC_3_F_3__RBS_sd14_S2_A (BC_3_F_3__203_A)	Backcross line – BC_3_F_3_	Large A09 (A09+)	0.09 ± 0.13 (ac)	9.87 ± 27.91 (b)

avg ± SD, average ± standard deviation. GI/g, galling index per gram of root. Eggs/g, number of eggs per gram of root. Columns with the same letter do not differ significantly (P < 0.05). Full data in Supplementary Table 8. *Resistance was derived from A. stenosperma, based on genotyping by Axiom_Arachis2 SNP array. ^**^ Resistance was derived from A. cardenasii.

Peanut root-knot nematode (PRKN) populations were cultured and extracted from eggplant (*Solanum melongena*) to be used as inoculum for bioassays. Second stage juveniles (J_2_s) were collected from infected roots in a mist chamber every 2–3 days over a week and stored at 10°C until inoculation. Peanut seeds were grown in nursery pots filled with steam-sterilized sandy soil in the greenhouse. Bioassay was performed under greenhouse conditions, in a randomized complete block design with 12 replicates per genotype. Furthermore, 40-day-old plants were inoculated with 6,000 J_2_s by adding the inoculum in two 2-cm deep holes at the base of the plant. Two months later, plants were uprooted, rinsed to remove soil, assessed for galling, and weighed. Eggs were extracted from roots using 0.5% NaOCl and counted (Hussey and Barker, 1973; Holbrook et al., 2003). Two different traits were measured: (1) galling index (GI), where 0 = no galls, 1 = 1-2 galls, 2 = 3-10 galls, 3 = 11-30 galls, 4 = 31-100 galls and 5 = more than 100 galls (Taylor and Sasser, 1978) and (2) number of eggs. Galling index and number of eggs per root weight (GI/g and eggs/g) were used for resistance assessment. A highly resistant plant was defined as such when the reproduction of nematodes was less than 20% of the reproduction in a susceptible plant (Taylor and Sasser, 1978).

### Phenotypic Characterization

#### Seed Size

In each cycle of backcrossing, 11 BC_1_F_1_, 30 BC_2_F_1_, 253 BC_3_F_1_, 101 BC_3_F_2_ and 25 BC_4_F_1_ seeds were phenotyped for weight (g), length (mm, longest point) and width (mm, widest point) prior to planting. For controls (*A. stenosperma* V10309; *A. batizocoi* K9484; BatSten1; *A. hypogaea* genotypes, 5-646-10, 13-1014, TifGP-2 and Runner-886), 10 individual seeds were measured (Table 2).

**TABLE 2:** Average and standard deviation for seed weight, length, and width measured in BC_n_F_n_ lines, diploid wild species, induced allotetraploid BatSten1, and cultivated controls.

Genotype	Type	Weight (g) min – max (Avg ± SD)	Length (mm) min – max (Avg ± SD)	Width (mm) min – max (Avg ± SD)	N
*A. stenosperma* V10309	Diploid wild species	0.16 – 0.19 (0.17 ± 0.01 b)	11.10 – 13.39 (11.86 ± 0.64 d)	4.93 – 5.57 (5.21 ± 0.21 c)	10
*A. batizocoi* K9484	Diploid wild species	0.14 – 0.31 (0.22 ± 0.05 c)	11.26 – 14.70 (12.95 ± 1.21 e)	5.01 – 6.37 (5.70 ± 0.43 d)	10
BatSten1	Induced allotetraploid	0.13 – 0.20 (0.17 ± 0.03 b)	11.18 – 14.45 (12.52 ± 0.92 e)	4.34 – 5.41 (5.05 ± 0.33 c)	10
*A. hypogaea* 5-646-10	Cultivated (Recurrent parent)	0.67 – 1.03 (0.81 ± 0.10 a)	14.30 – 20.22 (17.21 ± 1.87 ab)	9.71 – 11.20 (10.43 ± 0.51 ab)	10
*A. hypogaea* 13-1014	Cultivated (Recurrent parent)	0.58 – 0.97 (0.84 ± 0.11 a)	15.30 – 20.19 (18.04 ± 1.62 a)	8.50 – 11.54 (10.64 ± 0.89 a)	10
*A. hypogaea* TifGP-2	Cultivated (Recurrent parent)	0.59 – 0.91 (0.75 ± 0.12 a)	14.23 – 17.66 (16.48 ± 1.22 bc)	9.06 – 10.89 (9.81 ± 0.63 b)	10
*A. hypogaea* Runner-886	Cultivated control	0.51 – 0.94 (0.74 ± 0.17 a)	12.61 – 17.77 (15.07 ± 1.70 c)	8.40 – 11.26 (10.23 ± 1.01 ab)	10
BC_1_F_1_s	Backcross lines	0.29 – 1.46 (0.68 ± 0.34)	–	–	11
BC_2_F_1_s	Backcross lines	0.25 – 1.47 (0.72 ± 0.24)	11.01 – 21.46 (15.66 ± 2.15)	7.48 – 13.91 (9.87 ± 1.34)	30
BC_3_F_1_s	Backcross lines	0.09 – 1.31 (0.66 ± 0.25)	9.22 – 20.83 (15.60 ± 2.30)	3.71 – 13.42 (9.51 ± 2.04)	253
BC_3_F_2_s	Backcross lines	0.40 – 1.17 (0.72 ± 0.16)	13.47 – 22.05 (16.94 ± 1.93)	8.19 – 12.57 (10.11 ± 0.94)	101
BC_4_F_1_s	Backcross lines	0.42 – 0.97 (0.69 ± 0.13)	12.95 – 17.33 (15.83 ± 1.09)	7.96 – 12.32 (10.23 ± 1.06)	25

*Minimum and maximum values for controls and BC_n_F_1_ seeds are presented.*

*Weight, length, and width for controls with the same letter do not differ significantly (P < 0.05). Min-max (avg ± SD), minimum – maximum (average ± standard deviation) values are presented. N, number of seeds. Full data is in Supplementary Table 9.*

#### Pollen Viability (BC_2_F_1_s)

Pollen viability (PV) was evaluated for the BC_2_F_1_s and controls (Table 3). Flowers were collected early morning (between 8:00 and 10:00 am) and stained with acetocarmine (Heslop-Harrison, 1992). Stained pollen grains were observed and counted under a microscope (40X). Pollen viability from 10 individual flowers (reps) per genotype was assessed as the percentage of stained pollen grains (Gaaliche et al., 2013).

**TABLE 3:** Average and standard deviation for pollen viability (%) and the number of pods quantified in BC_2_F_1_s lines and controls.

Genotype (short)	Type	Pollen viability (%)	Number of pods
*A. stenosperma* V10309	Diploid wild species	94.90 ± 1.23 (b)	107
*A. batizocoi* K9484	Diploid wild species	71.69 ± 11.45 (cd)	260
BatSten1	Induced allotetraploid	75.51 ± 11.01 (c)	172
*A. hypogaea* 5-646-10	Cultivated (Recurrent parent)	93.63 ± 1.17 (ab)	120
*A. hypogaea* 13-1014	Cultivated (Recurrent parent)	90.24 ± 2.78 (ef)	95
BC_2_F_1_:C2633-2_3(16)	Backcross line – BC_2_F_1_	92.10 ± 3.46 (ae)	60
BC_2_F_1__BRD_C0049_Seed1 (BC_2_F_1__Seed1)	Backcross line – BC_2_F_1_	86.62 ± 5.60 (fg)	109
BC_2_F_1__BRD_C0049_Seed2 (BC_2_F_1__Seed2)	Backcross line – BC_2_F_1_	79.50 ± 4.81 (c)	48
BC_2_F_1__BRD_C0049_Seed7 (BC_2_F_1__Seed7)	Backcross line – BC_2_F_1_	80.84 ± 7.05 (cg)	5
BC_2_F_1__BRD_C0049_Seed8 (BC_2_F_1__Seed8)	Backcross line – BC_2_F_1_	65.08 ± 6.05 (d)	47
BC_2_F_1__BRD_C0050_Seed9 (BC_2_F_1__Seed9)	Backcross line – BC_2_F_1_	75.27 ± 5.19 (c)	103
BC_2_F_1__BRD_C0055_Seed15 (BC_2_F_1__Seed15)	Backcross line – BC_2_F_1_	Not evaluated	49
BC_2_F_1__BRD_C0055_Seed17 (BC_2_F_1__Seed17)	Backcross line – BC_2_F_1_	65.58 ± 9.14 (d)	107
BC_2_F_1__BRD_C0057_Seed28 (BC_2_F_1__Seed28)	Backcross line – BC_2_F_1_	89.81 ± 2.29 (ef)	260
BC_2_F_1__BRD_C0058_Seed33 (BC_2_F_1__Seed33)	Backcross line – BC_2_F_1_	77.90 ± 3.68 (c)	172

*Pollen viability (%) – Percentage of viable pollen grains. The pollen viability (%) column with the same letter does not differ significantly (P < 0.05). Number of pods – Total number of pods per plant. The correlation between Pollen viability (%) and the Number of pods was –0.007 (P < 0.05).*

#### Leaf Spot Incidence, Fertility, Architecture, and Flower Color (BC_3_F_1_s)

**FIGURE 2:**
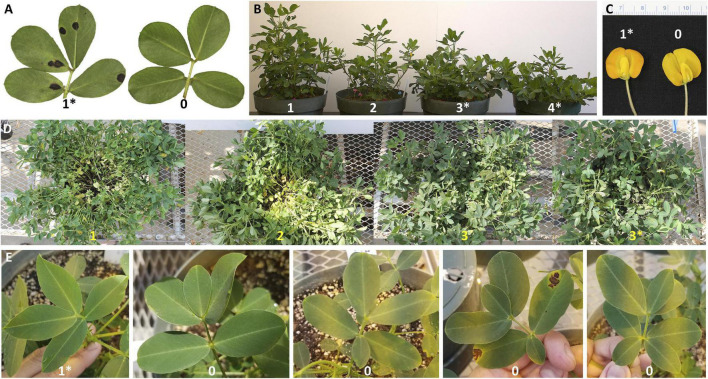
Phenotypic characterization of advanced backcross population. Leaf spot incidence for BC_3_F_1_s and BC_3_F_2_s [“yes” (1) or “no” (0)] **(A)**; architecture for BC_3_F_1_s and BC_3_F_2_s (scores: 1 – erect, 2 and 3 – intermediate, 4 – prostrate, and 5 – dwarf phenotype observed only for BC_3_F_2_ generation) (Pittman, 1995) **(B)**; flower color for BC_3_F_1_s (scores: Orange – 1 or yellow – 0) **(C)**; variation in branching for BC_3_F_2_s (scores: 1 – high, 2 – intermediate and 3 – normal) **(D)**; 1–3 extra leaves in leaflets for BC_3_F_2_s (score 0) **(E)**. “*” indicates *A. hypogaea* phenotype in all the cases.

While the BC_3_F_1_s were growing in the greenhouse, segregation for different traits was noticed, including foliar disease incidence, fertility (number of pegs), plant architecture, and flower color (Figures 2A–C). Single plant measurements were recorded. Leaf spot incidence was scored as a categorical variable as: “yes” (1) for *A. hypogaea* phenotype (susceptible) or “no” (0) for the resistant phenotype (Figure 2A). A total number of pegs was counted for assessment of fertility. Plant architecture or growth habit was scored from 1 to 4, with 1 being erect and 4 for prostrate growth habit (Figure 2B) (Pittman, 1995). Lastly, flower color was scored visually as orange (1) for *A. hypogaea* phenotype versus yellow (0) for the wild phenotype (Figure 2C).

#### Leaf Spot Incidence, Architecture, Branching, and Extra Leaves (BC_3_F_2_s)

A group of 101 BC_3_F_2_ plants from 12 BC_3_F_1_ families with a high recurrent parent (*A. hypogaea*) genome recovery (88.3–97.3%) were selected to create a group of lines carrying different sizes of introgressions in A02 and A09 for future gene cloning experiments. KASP assays targeting ten SNPs at A02-QTL and A09-QTL (Supplementary Table 2, gray-shaded markers) were used to confirm the presence of the QTL. While the plants were growing, several traits were recorded: leaf spot incidence and plant architecture were scored as above, in addition, a score of 5 for dwarf phenotype was included (Figures 2A,B). Branching was scored from an abnormal number of branches (high) (1) to intermediate (2) to normal *A. hypogea* phenotype (3) (see Figure 2D). Finally, extra leaves in leaflets were observed and scored as 1 for *A. hypogaea* and 0 for the presence of at least one extra leaf (Figure 2E).

### Association Analysis Using the BC_3_F_1_ Population

Association between genome-wide genotypic data of BC_3_F_1_ lines and phenotypic data of seed weight, length and width, leaf spot incidence, fertility, architecture, and flower color were used to identify candidate wild introgressions that could be controlling these traits. For seed size, leaf spot incidence, fertility, and architecture a Pearson correlation was performed. Flower color was analyzed using a mixed linear model (MLM) in Tassel (v.5) (Ithaca, NY, United States) (Bradbury et al., 2007). A Manhattan plot was created in R using the *“qqman”* package (Turner, 2014) and thresholds calculated using the *“CalcThreshold”* package with the Bonferroni method (Hamazaki and Iwata, 2020).

### Statistical Analysis

Phenotypic data for seed weight, length and width, pollen viability, and nematode resistance bioassay were analyzed using the package R. A Shapiro-Wilk test was used to test for normal distribution. Non-parametric Kruskal-Wallis one-way analysis of variance (Kruskal and Wallis, 1952) was used to evaluate differences at a 5% level of significance (*P* < 0.05). For the seed weight, length, and width (transformed to Log10 when needed) the Welch *t*-test was used to perform pair-wise comparisons between wild accessions, cultivated genotypes, and backcross generations. Additionally, the non-parametric Skillings-Mack test (Chatfield and Mander, 2009) was used to evaluate significant differences for the RCBD nematode bioassay (*P* < 0.05). Further analysis included the Wilcoxon signed-rank test for pairwise comparisons using false discovery rate (FDR) correction to group samples by significant similarity (*P* < 0.05).

## Results

### Marker-Assisted Breeding

Four generations of MABC for introgression of PRKN resistance from *A. stenosperma* were completed in two locations, Athens, GA, and Tifton, GA under greenhouse conditions (Figure 1). KASP genotyping was performed using 16 (for first, second and third cycles) and 10 SNP markers (fourth cycle) (Supplementary Tables 1, 2, gray-shaded markers). For the first cycle of backcrosses, 38 cross combinations were used, with 19 F_2:3_ plants as donor male parents and two cultivated peanut female parents, namely, TifGP-2 and 5-646-10. In this cycle, 1,008 potential BC_1_F_1_ seeds were harvested, and based on KASP genotyping, 17 were selected for harboring the nematode resistance segments in A02 and A09 (Supplementary Table 3).

For the second cycle, 14 cross combinations were made with four cultivated peanut female parents, TifGP-2, 5-646-10, 13-1014 and 13-2113, and 11 BC_1_F_1_ male parents. Here, 61 potential BC_2_F_1_ seeds were obtained and genotyped with KASP markers. A total of 21 were selected as they harbored resistance segments in A02 and A09 PRKN resistance QTL (Supplementary Table 4).

For the third cycle of backcrossing, a total of 21 cross combinations were made using 5-646-10 and 13-1014 as female parents and 21 BC_2_F_1_ male parents selected the previous year, six of which were shown to be resistant to PRKN (Ballén-Taborda et al., 2021). This resulted in 397 potential BC_3_F_1_ seeds (Supplementary Table 5), that were genotypically characterized in two different ways. First, 81 BC_3_F_1_ seeds were randomly selected and evaluated for the presence of resistance segments using the KASP assays. From this group, 52 BC_3_F_1_s harbored the segments in A02 and A09 from *A. stenosperma* (Supplementary Table 5). Second, 271 BC_3_F_1_s were genome-wide genotyped (see next section).

For the fourth cycle, six cross combinations were used with 5-646-10, 13-1014, and 13-1125 as females and two BC_3_F_2_ male parents harboring A02 and A09 loci. Here, 27 potential BC_4_F_1_ seeds were obtained and genotyped with KASP markers. A total of 25 were confirmed to harbor PRKN resistance chromosome segments at the bottom of both A02 and A09 (Supplementary Table 6). Three of them could have combined both sources of resistance in A02 from *A. stenosperma* and A09 from *A. cardenasii* present in parent 13-1125.

### Genome-Wide Genotyping of BC_3_F_1_s

To characterize the wild introgressions in the BC_3_F_1_ population, 271 lines and controls were genotyped with the Axiom_Arachis2 SNP array. A total of 930 informative polymorphic SNP markers, previously identified and assigned to A and B subgenomes (Ballén-Taborda et al., 2019), were recovered. Among these, 527 markers were located in A-subgenome (*A. stenosperma*-specific markers) and 403 to B/K-subgenome (*A. batizocoi*-specific markers). Of the 271 genotyped lines, 253 (93.4%) were true progeny from hybridization and 18 (6.6%) were products of self-pollination (Supplementary Table 7).

Examples of Axiom clustering plots of SNPs linked to QTL in A02 (Supplementary Figures 1A,B) and A09 (Supplementary Figures 1C–E) show the distribution of BC_3_F_1_ lines and controls. Red clusters (A02 SNPs) and blue clusters (A09 SNPs) comprise genotypes without the *A. stenosperma*-derived allele. *A. stenosperma* was genotyped with the alternate allele. Yellow clusters indicate the backcross lines with incorporated PRKN resistance from *A. stenosperma*.

**FIGURE 3:**
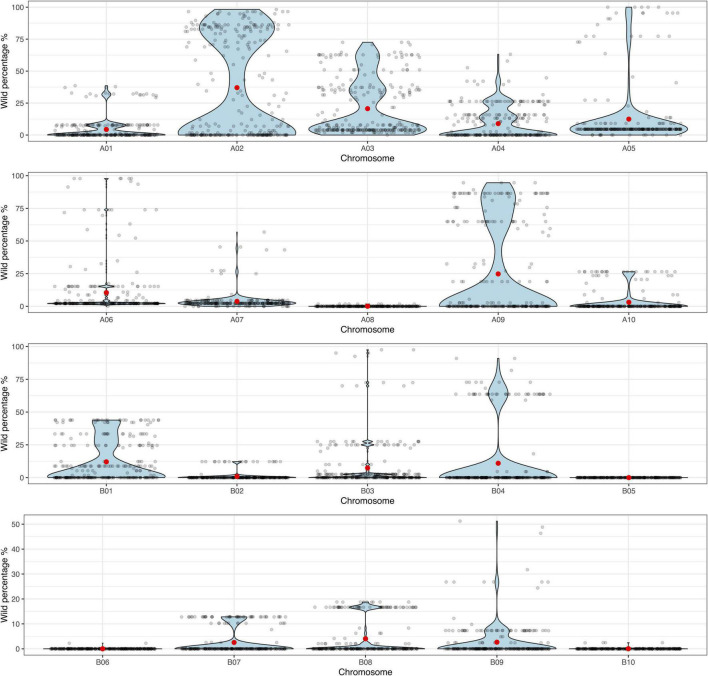
Violin plots for the proportion of wild genome (%) (y-axis) in each of the 10 A- and 10 B-subgenome chromosomes (x-axis) for the 271 BC_3_F_1_ lines. Black dots indicate each individual BC_3_F_1,_ and red dots indicate the mean.

The principal component analysis (PCA) performed on the genotyping data, allowed us to observe that the BC_3_F_1_s have recovered a high percentage of the *A. hypogaea* genome. Backcross lines showed a recurrent parent genome recovery between 80.2 and 98.8%, while still carrying between 1.1 and 19.1% of the wild donor genome (Supplementary Figure 2). Additionally, to visualize the distribution of the backcross population according to the proportion of wild introgression (%) in each chromosome, the data was displayed in violin plots. These plots allowed us to observe that the lines were harboring more wild alleles in the A-subgenome than in B-subgenome, especially in chromosomes A02 and A09 where foreground selection was applied during the backcrossing process (Figure 3 and Supplementary Table 7).

### Peanut Root-Knot Nematode Resistance Validation Using BC_3_F_3_s

To validate the PRKN resistance controlled by QTL in A02 and/or A09, 72 BC_3_F_3_ segregating lines, and resistant and susceptible controls were evaluated using a greenhouse pot nematode bioassay and genotyped with both KASP and Affymetrix to confirm the presence of the QTL. Specifically, Affymetrix was completed for genome-wide characterization. Galling index (GI) and number of eggs in relation to root weight (GI/g and eggs/g) allowed us to assess the resistance to *M. arenaria* within the backcross lines (Table 1).

**FIGURE 4:**
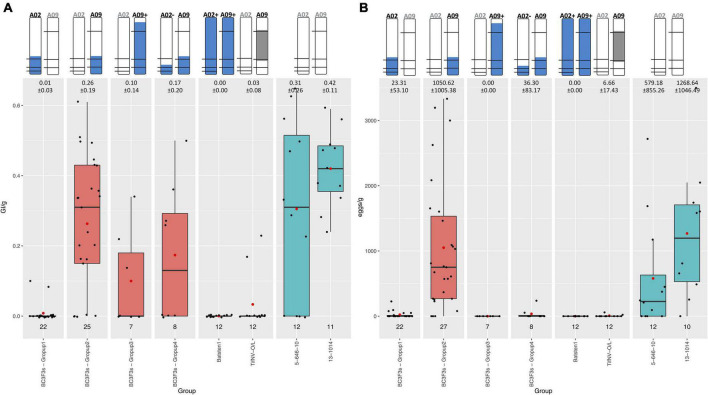
Boxplot diagrams for Galling index per gram of root (GI/g) **(A)** and Number of eggs per gram of root (eggs/g) **(B)** of BC_3_F_3_ lines, resistant controls Batsten1 and *A. hypogea* TifNV-High O/L, and susceptible controls 5-646-10 and 13-1014. BC_3_F_3_ lines were grouped according to the *A. stenosperma* alleles that they are carrying as: • Group 1: bottom A02 (A02) (*A. stenosperma* allele at A02-83,464,195, A02-92,077,207, and A02-92,983,792); • Group 2: bottom A09 (A09) (*A. stenosperma* allele at A09-112,309,231 and A09-114,515,959); • Group 3: large A09 (A09+) (*A. stenosperma* allele at A09-16,516,448, A09-112,309,231 and A09-114,515,959); and • Group 4: Both bottom small A02 (*A. stenosperma* allele at A02-92,077,207 and A02-92,983,792) and bottom A09 (A02- and A09). The top of the figure indicates the introgression in A02 and A09 from *A. stenosperma* in blue and SNP markers as black horizontal lines. For TifNV-High O/L, resistance from *A. cardenasii* is colored in gray. The numbers at the top of the bars indicate the mean ± *SD*. The numbers above the groups indicate the number of lines included in each group. Complete pedigree in Supplementary Table 8. Black bars across boxes indicate the median and red dot the mean. BC_3_F_3_s in salmon color and controls in teal color.

Resistant controls (BatSten1 and TifNV-High O/L) and susceptible genotypes (5-646-10 and 13-1014) exhibited the expected phenotype. BatSten1 and TifNV-High O/L showed strong resistance, with no or low gall/egg production. In contrast, for 5-646-10 and 13-1014, GI/g fluctuated between 0.31 ± 0.26 and 0.42 ± 0.11 and eggs/g varied between 579.18 ± 855.26 and 1268.64 ± 1046.49 (Figure 4, Table 1 and Supplementary Table 8). BC_3_F_3_ lines showed significant differences for GI/g and eggs/g (Kruskal-Wallis, Skillings-Mack and Wilcoxon tests, *P* < 0.05). Since the BC_3_F_3_s were still recombining and segregating for PRKN QTL, to better summarize the results, the lines were grouped according to the segments they were carrying as follows: • Group 1: bottom A02 (A02) (*A. stenosperma* allele at A02-83,464,195, A02-92,077,207 and A02-92,983,792 → 81.0 – 93.8 Mb); • Group 2: bottom A09 (A09) (*A. stenosperma* allele at A09-112,309,231 and A09-114,515,959 → 104.6 – 119.8 Mb); • Group 3: large A09 (A09+) (*A. stenosperma* allele at A09-16,516,448, A09-112,309,231 and A09-114,515,959 → 3.4 – 118.7 Mb); and • Group 4: Both bottom small A02 (*A. stenosperma* allele at A02-92,077,207 and A02-92,983,792 → 91.6 – 93.8 Mb) and bottom A09 (A02- and A09). According to this grouping, all the backcross materials belonging to groups 1, 3, and 4 exhibited high levels of resistance to PRKN. No or few galls (0.01 ± 0.03–0.17 ± 0.20) and low egg production (0.00 ± 0.00–36.30 ± 83.17) was observed in the infected roots. For groups 3 and 4 galls were observed in the roots, but the production of eggs was inhibited. In contrast, lines in group 2 were susceptible to PRKN by having GI/g and eggs/g values of 0.26 ± 0.19 and 1050.62 ± 1005.38, respectively (Figure 4). For more details of GI/g and eggs/g values for the BC_3_F_3_ lines, see Table 1 and Supplementary Table 8.

### Phenotypic Characterization

A wide variation of morphological and agronomic traits was observed in the backcross populations (BC_3_F_1_s and BC_3_F_2_s), including seed size, pollen viability, leaf spot incidence, fertility (number of pegs), plant architecture, flower color, branching, and extra leaves (Figure 2). Association analysis between phenotypic information and wild introgressions in the BC_3_F_1_ population was performed to identify candidate regions associated with these traits.

#### Seed Size

**FIGURE 5:**
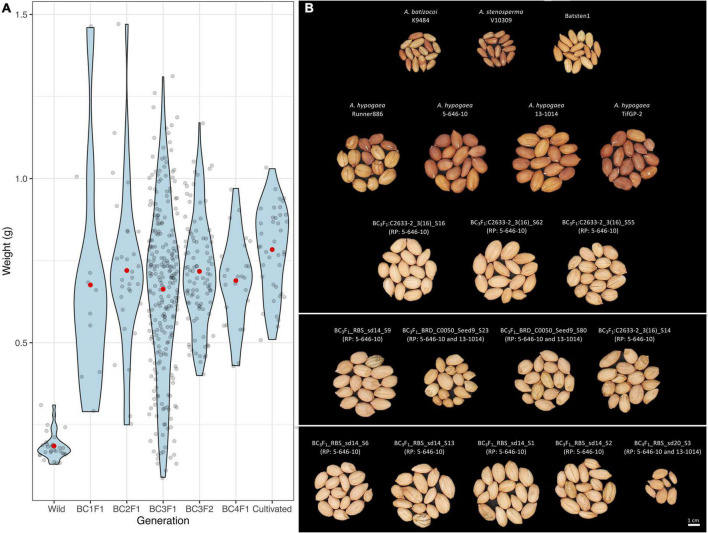
Violin plots for the distribution of seed weight (g) (y-axis) for wild controls, several backcross generations (BC_1_F_1_s, BC_2_F_1_s, BC_3_F_1_s, BC_3_F_2_s, and BC_4_F_1_s) and cultivated genotypes (x-axis); Black dots indicate each individual line and red dot the mean **(A)**. Photo of seeds of diploid species (*A. stenosperma* and *A. batizocoi*), synthetic allotetraploid BatSten1, cultivated genotypes (Runner-886, 5-646-10, 13-1014, TifGP-2), and seeds from BC_3_F_1_ lines **(B)**. Groups of 15 seeds are presented. Recurrent parent (RP) indicated in parenthesis for each BC_3_F_1_ line. Complete pedigree in Supplementary Table 7. The same trend for length (mm) and width (mm) was observed (Supplementary Table 9). Refer to Table 2 for average, *SD*, and statistical analysis for controls.

Seed weight (g), length (mm) and width (mm) measurements were recorded for 11 BC_1_F_1_, 30 BC_2_F_1_, 253 BC_3_F_1_, 101 BC_3_F_2_ and 25 BC_4_F_1_ seeds prior to planting, along with wild and cultivated controls. Significant differences were observed between the control genotypes according to the Kruskal-Wallis test and the Wilcoxon Test (*P* < 0.05), where wild genotypes have significantly smaller and lighter seeds as compared with seed dimensions of cultivated genotypes (Figure 5A, Table 2, and Supplementary Table 9). There was a clear recovery in seed size as early as BC_1_. According to the Welch *t*-test, the wild accessions (*A. stenosperma* V10309, *A. batizocoi* K9484, and BatSten1) differed significantly from the cultivated genotypes (*A. hypogaea* 5-646-10, 13-1014, TifGP-2 and Runner-886) and the backcross generations (BC_1_F_1_, BC_2_F_1_, BC_3_F_1_, BC_3_F_2_, and BC_4_F_1_) for weight, length, and width (*P* < 0.05). When comparing seed measurements of cultivated genotypes with each of the BC generations and between BC generations, in most of the cases there were no significant differences in seed size (*P* < 0.05 and *P* < 0.01) (Welch *t*-test matrix in Supplementary Table 9). Between the BC lines seed size exhibited variation, and on average the seed weight fluctuated from 0.66 g (BC_3_F_1_s) and 0.72 g (BC_2_F_1_s and BC_3_F_2_s), but having weight as high as 1.47 g when compared to the cultivated controls that exhibited similar seed weight of 0.74 g (Runner-886) and 0.84 g (13-1014), with the highest being 1.03 g. In this study, we presented data for the weight (Figure 5A), a similar tendency was observed for length and width (Table 2 and Supplementary Table 9). Based on the Pearson correlations performed between the seed weight, length, and width and the genotypic data for the BC_3_F_1_s potential *A. hypogaea* loci associated with large seed size were identified in chromosomes A03, B01, and B08 (Supplementary Figures 3A–C and Supplementary Table 10).

#### Pollen Viability (BC_2_F_1_s)

Pollen viability (PV) of wild accessions *A. stenosperma*, *A. batizocoi*, the induced allotetraploid BatSten1, recurrent parents 5-646-10 and 13-1014 and 9 BC_2_F_1_ lines was evaluated to estimate differences in viability within BC lines. Individuals showed varying degrees of pollen viability, ranging from 65.6 to 94.9% (Table 3). In control genotypes, PV varied from 71.7 to 94.9% and in the BC_2_F_1_s, it fluctuated from 65.6 to 89.8%. Although significant differences were observed between the genotypes according to the Kruskal-Wallis test and the Wilcoxon Test (*P* < 0.05), no grouping trend was observed. In summary, high pollen viability in the cultivated genotypes (5-646-10 and 13-1014), *A. stenosperma*, and some BC_2_F_1_s were observed, and lower pollen viability was observed for BatSten1, *A. batizocoi*, and other BC_2_F_1_s (Supplementary Figure 4). A low correlation between pollen viability and the number of produced pods per plant was observed (–0.007, *p* < 0.05).

#### Leaf Spot Incidence, Fertility, Architecture, and Flower Color (BC_3_F_1_s)

For the BC_3_F_1_ population that included 253 lines, segregation for leaf spot incidence, fertility, architecture, and flower color was noticed while growing in the greenhouse in a randomized position (Figures 2A–C). 150 lines exhibited signs of leaf spot, while 103 did not (Supplementary Figure 5A). Pearson correlation performed between phenotypic and genotypic data allowed us to identify two candidate loci in chromosomes A06 and B02 that could be associated with resistance to leaf spot (Supplementary Figure 3D) and are now being tested using BC_3_F_2_ progeny (data not shown). The number of pegs was counted as an indication of fertility. The distribution of the data showed that the majority of the lines had a similar number of pegs as the recurrent parents 5-646-10 and 13-1014, with an average of 13.15 ± 7.83 total number of pegs (Supplementary Figure 5B). For this trait, *A. hypogaea* loci in chromosomes A02 and A09 could be associated with fewer pegs (Supplementary Figure 3E). For architecture, around two-thirds of the lines had the cultivated growth habit phenotype (163) and one-third of the lines were exhibiting an erect growth habit (84) (six were too small to be scored) (Figure 2B and Supplementary Figure 5C). According to the association analysis, SNPs in chromosomes A01 and B08 could be associated with changes in plant architecture (Supplementary Figure 3F). Most lines exhibited orange flowers (245) while eight had the yellow color, a common trait within *Arachis* wild species (Supplementary Figure 5D). After running the mixed linear model in Tassel, introgression on the top of chromosome A05 (7.92–8.62 Mb) was found to be associated with the change in flower color from orange to yellow. In this study, four markers were found to be linked with this trait (A05-7,919,003, A05-7,958,564, A05-8,040,921, A05-8,621,849) (Supplementary Figure 6).

#### Leaf Spot Incidence, Architecture, Branching, and Extra Leaves (BC_3_F_2_s)

Segregation for leaf spot incidence, architecture, branching, and extra leaves was also noticed among the 101 BC_3_F_2_ lines (two of them were too small to be scored) (Supplementary Table 11). 38 lines had leaf spots and 61 did not (Supplementary Figure 5E). Around half of the lines had the cultivated growth habit phenotype (51), some exhibiting an erect phenotype (41) and a few with a dwarf phenotype (7) (Supplementary Figure 5F). Most lines showed the peanut phenotype of normal branching (79 with score 3) and some presented a high number of stems (20 with score 1 or 2) (Supplementary Figure 5G). Finally, at least one extra leaflet was observed in 70 plants (score 0) and 31 did not display this unexpected phenotype (score 1) (Supplementary Figure 5H).

## Discussion

Crop wild relatives (CWR) have become an important source to reintroduce genetic diversity for crop improvement (Dempewolf et al., 2017). For peanut breeding, diploid wild relatives comprise a diverse genetic pool that is being used to broaden peanut’s genetic base (Stalker, 2017). Transferring wild beneficial alleles requires an additional step of developing peanut compatible wild-derived synthetic allotetraploids (Suassuna et al., 2020). To incorporate root-knot nematode resistance from *A. cardenasii*, synthetic allotetraploids were successfully developed and used (Simpson et al., 1993; Simpson and Starr, 2001). The introgression in A09 controlling PRKN resistance (Nagy et al., 2010; Chu et al., 2016) is present in several commercial cultivars (Simpson et al., 2003, 2013; Holbrook et al., 2008, 2017; Branch and Brenneman, 2015) and has provided a strong resistance over the years. Since the resistance to PRKN is controlled by a single source, there is a risk of virulent nematode populations developing. Therefore, the incorporation of new alleles is essential to provide stronger and more durable resistance against PRKN.

One of the peanut wild relatives *A. stenosperma* PI666100/V10309 has been confirmed to be resistant to peanut root-knot nematode, *M. arenaria* (Proite et al., 2008), and genes involved in plant defense against this pathogen have been described (Proite et al., 2007; Guimarães et al., 2010; Mota et al., 2018; Araujo et al., 2021). The present work reports the successful incorporation of two new and strong PRKN resistance loci from *A. stenosperma* previously mapped and validated (Leal-Bertioli et al., 2016; Ballén-Taborda et al., 2019, 2021). Here, marker-assisted backcross breeding was employed to complete four cycles, and genetic and phenotypic characterization was performed (Figure 1). As pyramiding of major R-genes has been proven to be valuable to extend durability and effectiveness of major genes (Pilet-Nayel et al., 2017), in the fourth cycle the elite breeding line 13-1125 harboring nematode resistance from *A. cardenasii* was included as a female parent (Holbrook, CC, unpublished data). Based on KASP genotyping, three BC lines could have pyramided both sources of resistance in A02 from *A. stenosperma* and A09 from *A. cardenasii* (Supplementary Tables 6, 12, highlighted in green).

A population of 253 true third backcross lines were subjected to genome-wide genotyping and phenotypically characterized for association analysis. These lines originally selected for PRKN resistance had wild introgressions between 1.1 and 19.1% across the genome. Having a high percentage of the wild genome would indicate that additional cycles of backcrossing are needed to assure maximum elite genome representation; therefore, we completed the fourth cycle. However, segregation for several phenotypic attributes (seed size, pollen viability, leaf spot incidence, fertility, architecture, flower color, branching, and extra leaves) was observed, which indicated that wild introgressions in different chromosomes could be controlling these traits and are worth further study. The whole BC_3_ population has been carried forward not only to develop nematode-resistant cultivars but additionally to study resistances to other pests and diseases and to develop a CSSL-like population that would be useful for precise mapping of QTL.

Based on the data of agronomic and morphological traits measured in several generations, most lines exhibited elite peanut traits and were similar to recurrent parental breeding lines. First, seed size in each generation of introgression was examined. This was done to observe the seed size recovery as we progressed through our MABC scheme, as seed size is an important trait associated with germination, vigor, and yield, and is important for the peanut industry and market (Singh et al., 1998). For the backcross lines, our measurements showed that seed size was not progressively increased as the wild genome representation was reduced in each generation. On the contrary, we observed that only one backcross generation was required to recover the elite peanut’s large seeds and that in later generations the average seed size did not change significantly (Figure 5B). We also observed that larger and heavier seeds were produced by some BC lines, by having seed weight as high as 1.47 g compared to the cultivated controls that exhibited 1.03 g as the heaviest seed. Similar behavior was observed for length and width. An explanation for the transgressive segregation in seed size could be due to the presence of wild alleles that are contributing to larger seed size, as reported before in interspecific peanut progenies (Fonceka et al., 2012a; Suassuna et al., 2015). Although the correlation analysis showed candidate regions in chromosomes A03, B01, and B08, this requires further validation as these have not been reported previously.

Results for pollen viability measured in the BC_2_ generation agreed with that of Leal-Bertioli et al. (2015) as cultivated peanut genotypes and *A. stenosperma* showed a high pollen viability (average of 91.9%) and most of the BC_2_F_1_ lines had lower numbers (average of 79.2%) reflecting the genetic distance of the parental genotypes. Our results showed little variation regardless of the genotype, which suggests that pollen viability is not a key contributor to the production of seeds within the backcross lines (Leal-Bertioli et al., 2015). This was also corroborated by the low correlation between pollen staining and pod production (–0.007, P < 0.05). The correlation analysis on the BC_3_ generation, also allowed us to identify candidate introgressions for leaf spot reduction in chromosomes A06 and B02. For flower color, the region at the top of chromosome A05 (7.92 – 8.62 Mbp) associated with the yellow color trait is consistent with previous reports, and a result of homologous recombination (Fonceka et al., 2012b; Bertioli et al., 2019).

Although most of the BC lines had domesticated features, we also observed some variation in fertility (number of pegs), plant architecture, branching, and extra leaves. Further analyses will be required to fully understand the candidate wild introgressions that are controlling these traits. Finally, in the case of extra leaflets, this is a phenotype that has been described as a novel heterozygous trait that continues to segregate even after several generations of selfing (Branch et al., 2020).

### Validation of Nematode Resistance

In this study, resistance to PRKN was successfully validated in a set of BC_3_F_3_ lines. The bottom of A02 (81.0 – 93.8 Mb) (Figure 4, group 1) provided strong resistance as previously described and validated (Leal-Bertioli et al., 2016; Ballén-Taborda et al., 2019, 2021). In the case of the QTL in chromosome A09, we observed that the small introgression at the bottom (104.6 – 119.8 Mb) was insufficient to stop nematode development (group 2) and that a larger segment at the top-bottom of A09 (3.4 – 118.7 Mb) was required to provide resistance, especially for preventing eggs production (group 3). It is possible that the presence of wild alleles in A04 associated with susceptibility in group 2 could be acting against resistance as previously described (Ballén-Taborda et al., 2019). When the plants were harboring both bottom small A02 (91.6 – 93.8 Mb) and bottom A09 (104.6 – 119.8 Mb) (group 4) we would expect to observe inhibition of both galls and egg production since A02 was present; however, galls were present in the roots. Field testing is in progress to test the stability of the resistance and for allele fixation through selfing.

### Implications for Breeding for Disease Resistance

Genetic maps, quantitative trait loci (QTL), and marker-phenotype associations have been reported for numerous crops and traits (Collard and Mackill, 2008). Despite this, examples of QTL incorporation in plant breeding programs are lower than expected (Bernardo, 2016). This work represents a successful example of QTL introgression from a wild relative into an elite peanut despite the genetic incompatibilities. This provides an alternative to the only source of root-knot resistance currently deployed in the peanut crop, derived from *A. cardenasii* (Simpson and Starr, 2001).

The population of advanced peanut backcross lines that we have developed during this work has wild chromosome segments through much of the genome, distributed in different ways in different lines. They are being tested and advanced in several locations, and the best performing lines are being selected for germplasm release. The PRKN resistance alleles have been successfully validated and DNA markers are now available to facilitate the marker-assisted selection. Furthermore, because of the diverse wild chromosome segments in this population, we also anticipate that it has other disease resistances and traits of value to the peanut crop. We anticipate that, over time, these backcrossed lines will impact peanut production by delivering several new traits to the peanut crop, similar to the case of North Carolina peanut lines with *A. cardenasii* segments that have provided resistance to late leaf spot, rust, and web blotch in numerous countries around the world (Bertioli et al., 2021b).

## Data Availability Statement

The original contributions presented in the study are included in the article/Supplementary Material, further inquiries can be directed to the corresponding author.

## Author Contributions

SCML-B, DJB, SAJ, and PO-A conceptualized this project. PO-A, YC, CCH, and CB-T performed crosses. CB-T and PT performed phenotyping for nematode resistance. CB-T performed phenotypic characterization of traits and wrote the original draft. CB-T, DJB, and SCML-B conducted genotyping and data analysis. All authors revised and approved the manuscript.

## Conflict of Interest

The authors declare that the research was conducted in the absence of any commercial or financial relationships that could be construed as a potential conflict of interest.

## Publisher’s Note

All claims expressed in this article are solely those of the authors and do not necessarily represent those of their affiliated organizations, or those of the publisher, the editors and the reviewers. Any product that may be evaluated in this article, or claim that may be made by its manufacturer, is not guaranteed or endorsed by the publisher.

## Footnotes

1
https://biosearch-cdn.azureedge.net/assetsv6/KASP-genotyping-chemistry-User-guide.pdf

2
https://wheat.pw.usda.gov/demos/BatchPrimer3/
