# Supplementary material for: Development and Genetic Characterization of Peanut Advanced Backcross Lines That Incorporate Root-Knot Nematode Resistance From Arachis stenosperma
Source: Front Plant Sci. 2022 Jan 17;12:785358. doi: 10.3389/fpls.2021.785358 (PMC8801422; doi:10.3389/fpls.2021.785358)
Supplement: Supplementary file 1 [file Data_Sheet_1.PDF]

Development and genetic characterization of peanut advanced backcross lines that incorporate root-knot nematode resistance from *Arachis stenosperma*

Carolina Ballén-Taborda, Ye Chu, Peggy Ozias-Akins, C. Corley Holbrook, Patricia Timper, Scott A. Jackson, David J. Bertioli and Soraya C.M. Leal-Bertioli

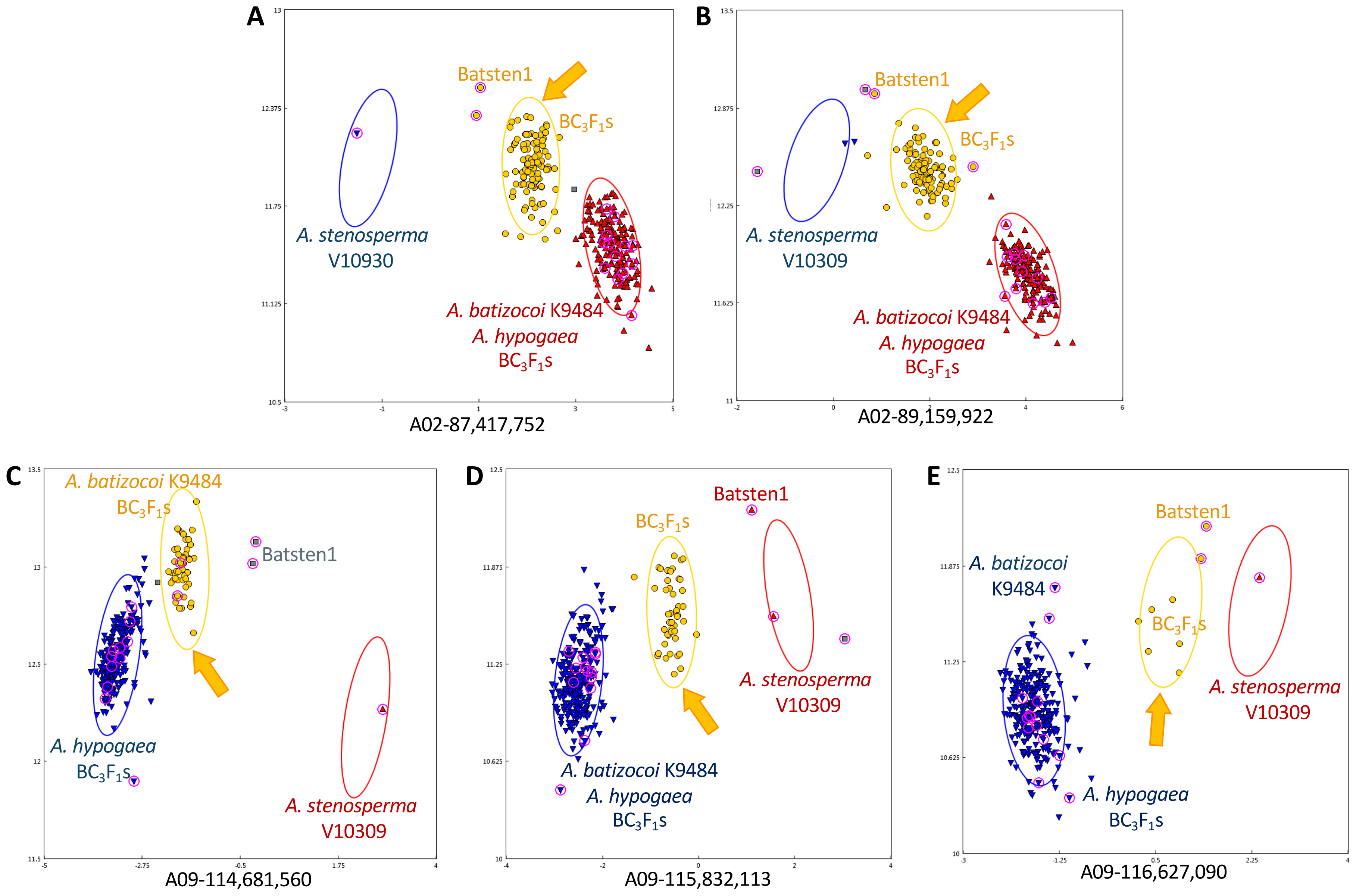

Figure S1. Screenshots of SNPs associated with nematode resistance from *A. stenosperma* in chromosome A02 **(A,B)** and A09 **(C,D,E)**. True backcross lines inside yellow clusters (indicated by arrows).

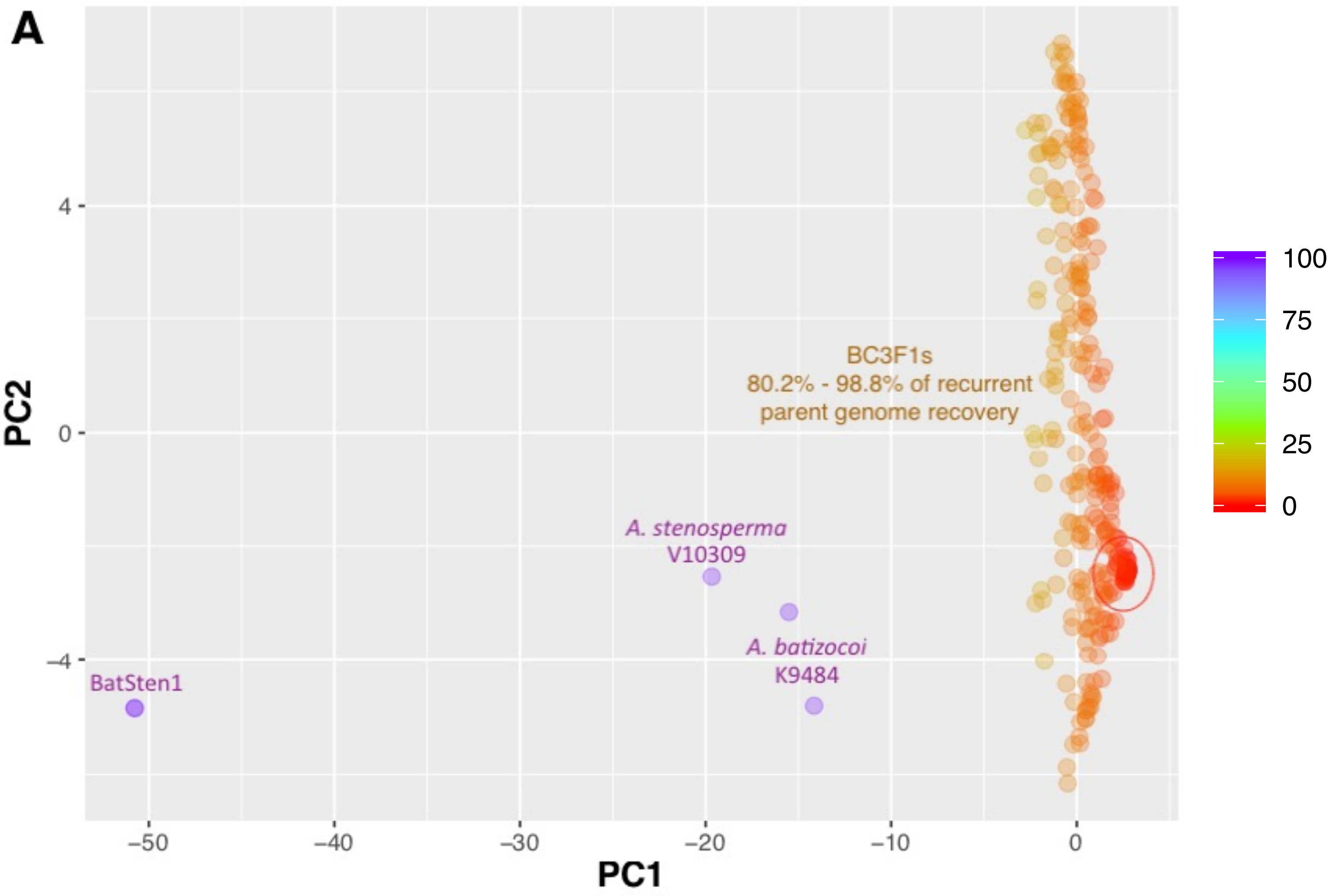

Figure S2. Principal components analysis (PCA) for 271 BC_3F_1 lines (orange cluster with percentage of *A. hypogaea* genome), diploid controls *A. stenosperma*, *A. batizocoi*, the synthetic allotetraploid BatSten1 (purple dots) and *A. hypogaea* genotypes (red cluster). The legend on the right side indicates the percentage of wild genome.

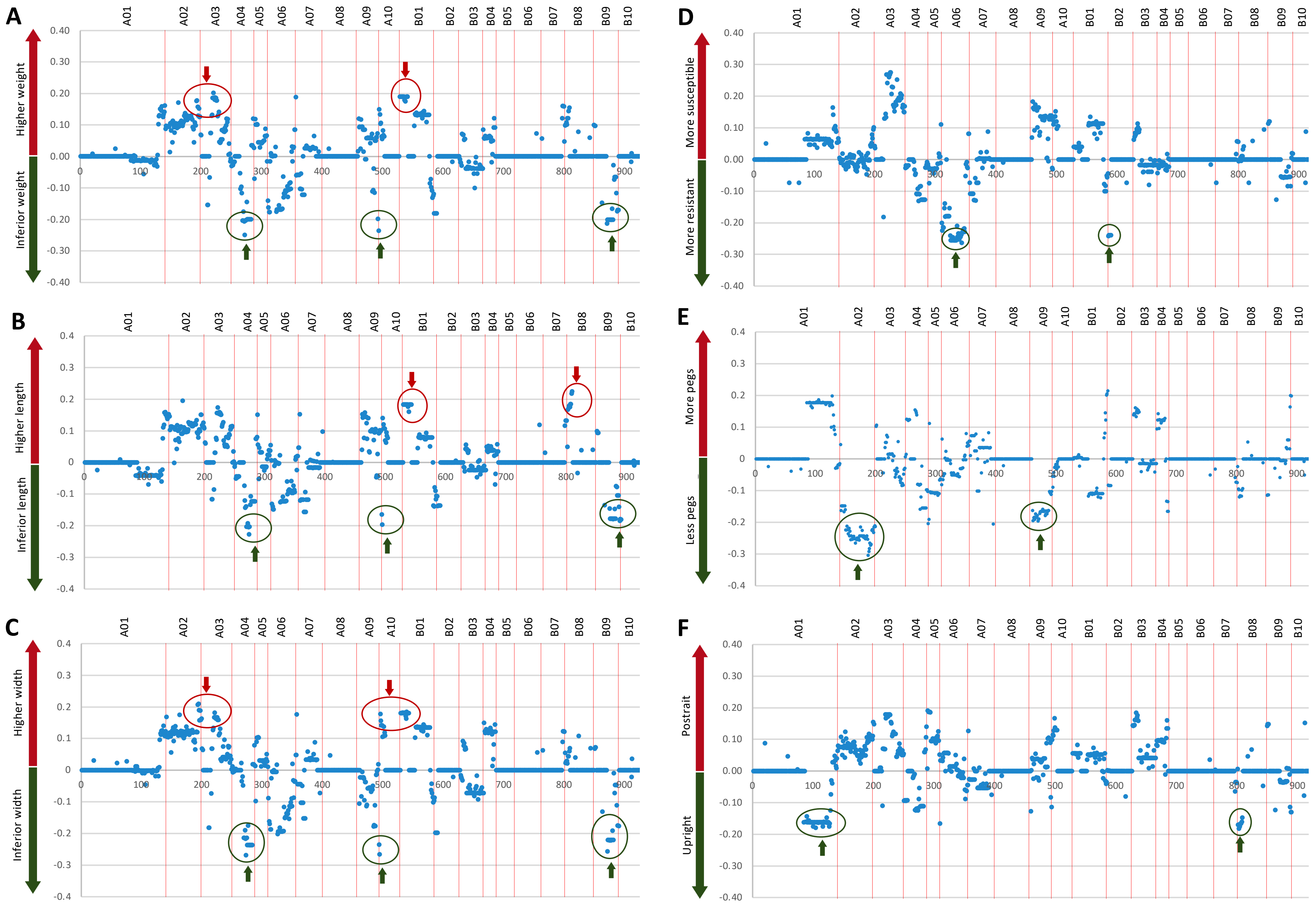

Figure S3. Pearson correlation plots between phenotypic and genotypic data (BC_3F_1 lines) for seed weight (g) **(A)**, length (mm) **(B)**, width (mm) **(C)**, leaf spot incidence **(D)**, total number of pegs **(E)** and architecture **(F)**.

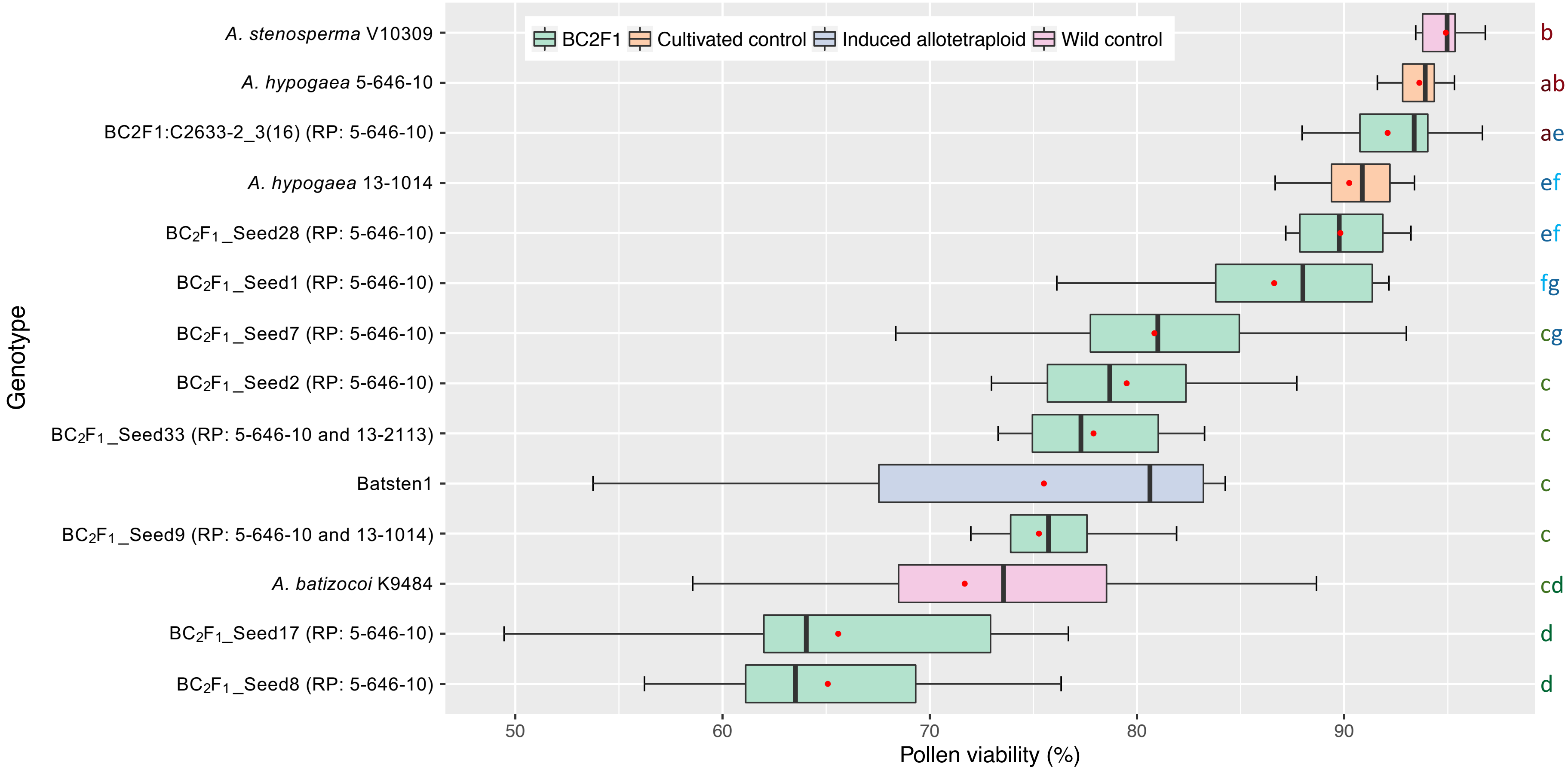

Figure S4. Boxplot diagram of estimated pollen viability (%) for 9 BC_2F_1 lines (light green), cultivated controls (light orange), induced allotetraploid BatSten1 (light blue) and wild accessions *A. stenosperma* and *A. batizocoi* (light pink). Boxes with the same letter do not differ significantly (P<0.05). Black bars across boxes indicate the median and red dot the mean. Left and right ends represent the highest and lowest values. Recurrent parent (RP) indicated in parenthesis for each BC_2F_1 line. Complete pedigree is described in previous report (Ballén-Taborda et al. 2021).

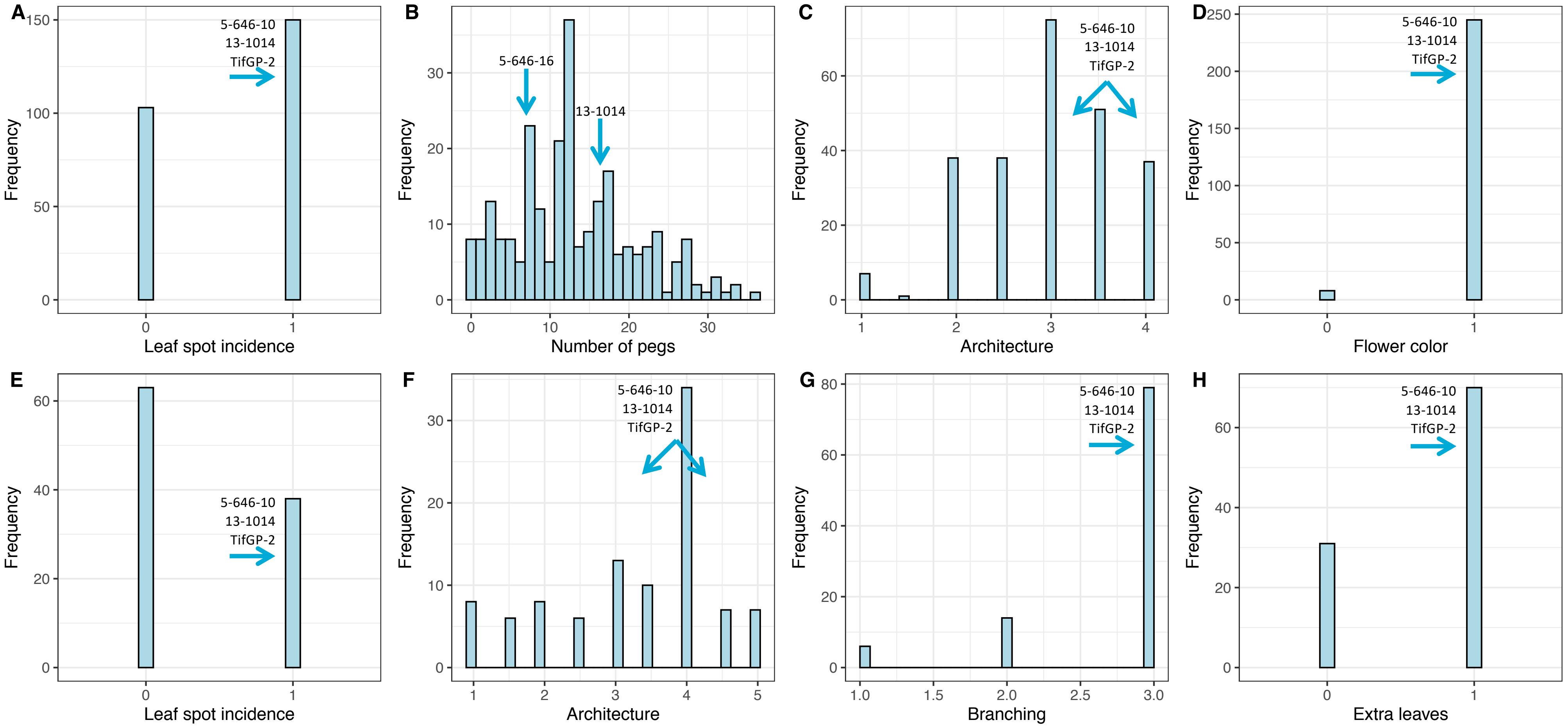

Figure S5. Frequency distribution for agronomic and morphological traits. Histograms for leaf spot incidence **(A)**, fertility (number of pegs) **(B)**, architecture **(C)** and flower color **(D)** evaluated in the BC_3F_1 population. Leaf spot incidence **(E)**, architecture **(F)**, branching **(G)** and extra leaves **(H)** measured in the BC_3F_2 generation. In each case phenotype for *A. hypogaea* 5-646-10, 13-1014 and TifGP-2 is indicated by arrow.

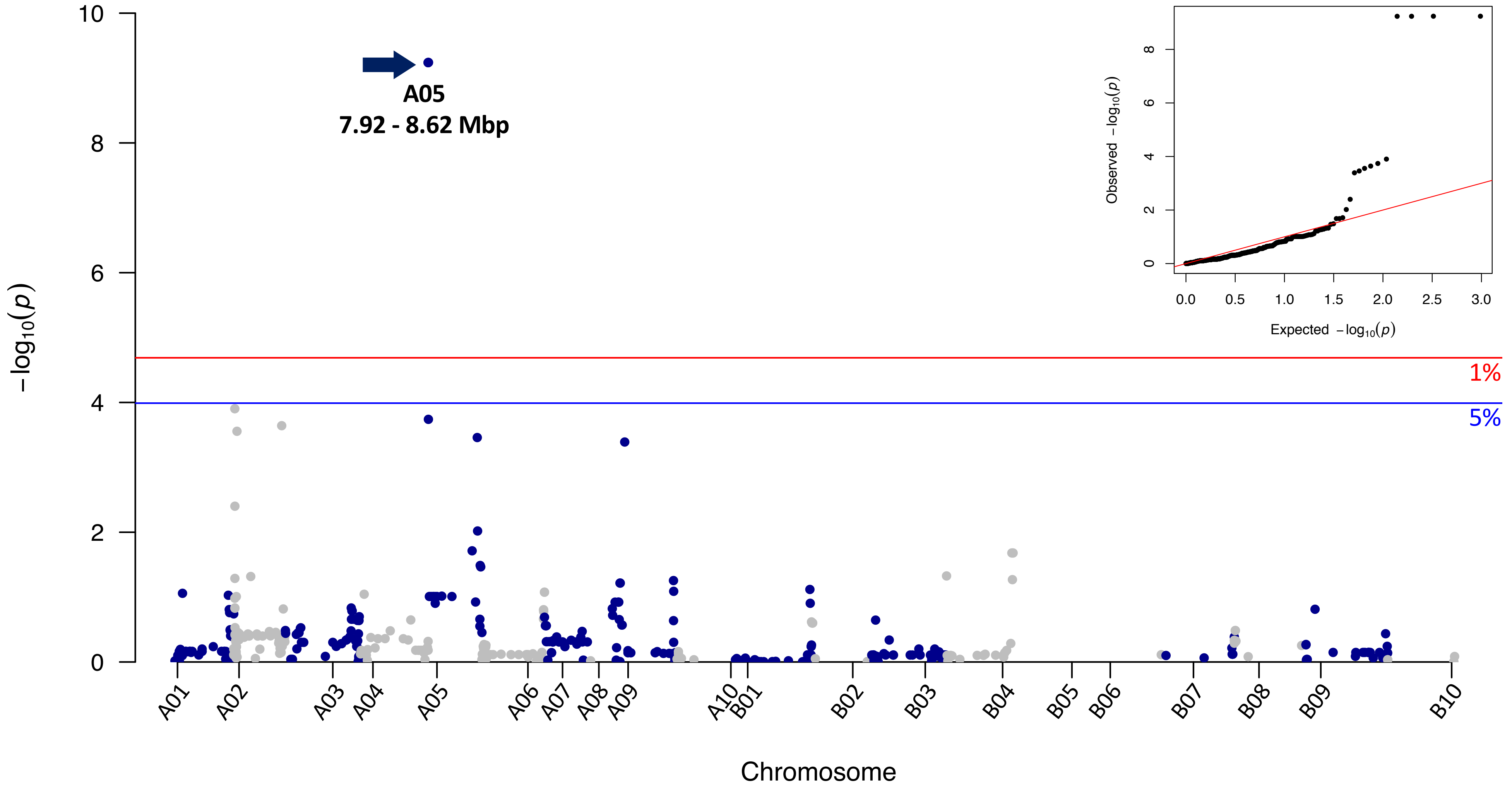

Figure S6. Manhattan plot for flower color. Negative $\log_{10}$ P-values in y-axis and chromosomes in x-axis. Thresholds at 1% (P<0.01) indicated in red and 5% (P<0.05) in blue. Q-Q plot at the top-right corner.
